# Evaluation of WIMU Sensor Performance in Estimating Running Stride and Vertical Stiffness in Football Training Sessions: A Comparison with Smart Insoles

**DOI:** 10.3390/s24248087

**Published:** 2024-12-18

**Authors:** Salvatore Pinelli, Mauro Mandorino, Mathieu Lacome, Silvia Fantozzi

**Affiliations:** 1 Department for Life Quality Studies, University of Bologna, 47921 Corso D’Augusto 237, 47921 Rimini, Italy; salvatore.pinelli2@unibo.it; 2 Performance and Analytics Department, Parma Calcio 1913, 43124 Parma, Italy; mlacome@parmacalcio1913.com; 3 Department of Movement, Human and Health Sciences, University of Rome “Foro Italico”, 00135 Rome, Italy; 4 Sport Expertise and Performance Laboratory, French National Institute of Sports (INSEP), 75012 Paris, France; 5 Department of Electrical, Electronic, and Information Engineering “Guglielmo Marconi”, University of Bologna, 40127 Bologna, Italy; silvia.fantozzi@unibo.it

**Keywords:** temporal parameters, vertical stiffness, inertial measurement units, running test

## Abstract

Temporal parameters are crucial for understanding running performance, especially in elite sports environments. Traditional measurement methods are often labor-intensive and not suitable for field conditions. This study seeks to provide greater clarity in parameter estimation using a single device by comparing it to the gold standard. Specifically, this study aims to investigate how the temporal parameters and vertical stiffness (K_vert_) of running stride exerted by IMU sensors are related to the parameters of the smart insole for outdoor acquisition. Ten healthy male subjects performed four 60-meter high-speed runs. Data were collected using the WIMU PRO™ device and smart insoles. Contact time (CT) and flight time (FT) were identified, and K_vert_ was calculated using Morin’s method. Statistical analyses assessed data normality, correlations, and reliability. WIMU measured longer CT, with differences ranging from 26.3% to 38.5%, and shorter FT, with differences ranging from 27.3% to 54.5%, compared to smart insoles, across different running speeds. K_vert_ values were lower with WIMU, with differences ranging from 23.96% to 45.01% depending on the running activity, indicating significant differences (*p* < 0.001). Using these results, a multiple linear regression model was developed for the correction of WIMU’s K_vert_ values, improving the accuracy. The improved accuracy of K_vert_ measurements has significant implications for athletic performance. It provides sports scientists with a more reliable metric to estimate player fatigue, potentially leading to more effective training regimens and injury prevention strategies. This advancement is particularly valuable in team sports settings, where easy-to-use and accurate biomechanical assessments of multiple athletes are essential.

## 1. Introduction

Temporal parameters play a pivotal role in understanding sprint running performance, particularly for track and field coaches. These parameters, such as the duration of the flight and stance phases, have been extensively explored in the literature and provide valuable insights into athletic performance. In football, physical performance encompasses a range of activities, including long-distance running, high-intensity bursts, and short sprints. These physical demands directly influence the technical skills that are essential for both individual players and overall team success [1]. Factors like strength, power, technique, and movement speed all contribute to a player’s physical performance [2]. To further understand human running performance, the spring-mass model was widely adopted in research [3,4,5,6]. This model posits that the body’s potential elastic energy is influenced by vertical stiffness (K_vert_), a key parameter for optimizing locomotion [7,8]. It is a quantitative measure of the body’s ability to absorb and restore potential elastic energy, largely governed by musculotendinous structures [3]. Maintaining sufficient Kvert during running allows athletes to perform more efficiently by minimizing vertical center of mass (COM) displacements and maximizing the return of elastic energy from these tissues. Morin et al. [9] proposed a model based on a point mass supported by a linear “leg spring”, offering a simplified yet effective means for assessing stiffness parameters.

Various technologies are used to monitor these biomechanical parameters, such as force platforms [10], stereophotogrammetric systems [11], optical bars [12], and video analysis [13,14]. However, these methods are often labor-intensive and time-consuming, making them impractical for elite environments where quick and efficient evaluations are required. Recently, wearable inertial measurement units (IMUs) have emerged as non-invasive tools for in-field performance monitoring. IMUs allow athletes to move freely during extended sessions, providing coaches with data that can help tailor individual and group training strategies in real-time [15,16]. Their integration into athlete training routines is increasingly vital in modern sports, aiding coaches in tailoring individual and group training strategies effectively, especially in team sports. Successful teams, like the English Premier League football teams, are now recognized to rely on multifactorial training regimens that take into account the intricate physiological demands of professional football [17]. IMUs have been successfully used to estimate temporal parameters during walking and running by identifying specific events in the acceleration signals collected from a trunk-mounted sensor [18,19]. These temporal parameters are closely linked to mechanical stiffness properties, which are critical for dynamic performance [20]. Recently, a neuromuscular status monitoring protocol was developed, specifically for field training, and validated with elite rugby and football players. It uses velocity and acceleration data collected during running [21,22]. In addition to IMUs, smart insoles have gained attention for their ability to provide detailed gait analysis in a portable and non-restrictive manner. Loadsol smart insoles have been validated in multiple studies for their reliability and accuracy in measuring load-related and spatio-temporal metrics during various functional activities. Specifically, K. E. Renner et al. [23] demonstrated that smart insoles deliver reliable and accurate measurements of peak weight acceptance force, loading rate, and impulse under various walking and running conditions, as indicated by high intra-class correlation (ICC) values. Cudejko et al. [24] have shown that Loadsol insoles exhibit good-to-excellent agreement with force platforms and instrumented treadmills, further confirming their robustness and effectiveness in running analysis. Likewise, high reliability and validity were established for the XSENSOR X4 insole system in the measurement of vertical ground reaction forces (GRFs) and center of pressure trajectories during hopping, walking, and running [25].

While smart insoles represent a significant advancement over traditional systems like force platforms and stereophotogrammetric systems, they are not ideal for monitoring entire teams due to the complexity of managing multiple devices simultaneously during training sessions. This is due to the need for numerous personnel and devices to be managed simultaneously during the entire training session. Given the practical challenges of using smart insoles in everyday training, IMUs offer a more feasible and practical alternative for everyday training environments.

In team sports, integrating IMUs with GPS sensors has become a widely adopted practice, as this combination delivers more comprehensive and detailed performance metrics. The scalability of these systems ensures their applicability across large teams, making them indispensable tools in modern sports science. The WIMU sensor is a tool that was previously validated for its accuracy and reliability [26,27,28,29] in estimating player positioning and parameters related to the magnitude of acceleration (e.g., PlayerLoad). However, it should be noted that this validation does not extend to the estimation of K_vert_, which has not been specifically assessed in the existing literature. Although wearable IMUs are widely adopted in sports science, their ability to estimate biomechanical parameters such as vertical stiffness (K_vert_) has not been thoroughly validated. This study aims to address this gap by systematically comparing the IMU with validated smart insoles under field conditions.

Due to the previously discussed limitations of smart insoles, this study seeks to provide greater clarity in parameter estimation using WIMU devices by comparing them to the gold standard. Specifically, this study aims to investigate how the temporal parameters and vertical stiffness of running stride exerted by IMU sensors are related to the parameters of the smart insole for outdoor acquisition. This comparison evaluates the accuracy and reliability of IMUs, providing insights into their potential as simpler and more accessible tools for monitoring biomechanical parameters.

## 2. Materials and Methods

### 2.1. Participants

Ten healthy male subjects were included in the study. The sample consisted of former professional athletes who had previously competed at a high level, with significant training experience and a solid understanding of intense physical activity dynamics. The study sample comprised participants with a mean age of 32.9 years (standard deviation (SD) = 6.1). The mean body mass of the participants was 80 kg (SD = 10.6), with a range between 61 and 97 kg. The mean height was 176.6 cm (SD = 5.8). Prior to data collection, all participants were briefed on the study protocols, including the potential risks involved. This study includes human participants and was approved by the University of Bologna Bioethics Committee (protocol No. 0351259). Informed consent was obtained from each participant prior to their involvement, and all procedures adhered to the approved protocol. In order to maintain the subjects’ privacy, all data were anonymized before the analysis, in accordance with the principles of the Declaration of Helsinki.

### 2.2. Experimental Setup

All subjects performed a tempo box-to-box run. The test consisted of four paced, high-speed runs. Each run was 60 meters long and the players had to complete the run in 12 seconds (average speed ≈ 18 km/h) with 33 seconds of recovery between trials, as suggested in previous research [22].

### 2.3. Data Collection

Physical data were collected using the WIMU PRO device (RealtrackSystems S.L., Almeria, Spain), a tool previously validated for its accuracy and reliability [26,27,28,29]. However, it should be noted that this validation does not extend to the estimation of K_vert_, which has not been specifically assessed in the existing literature.

**Figure 1:**
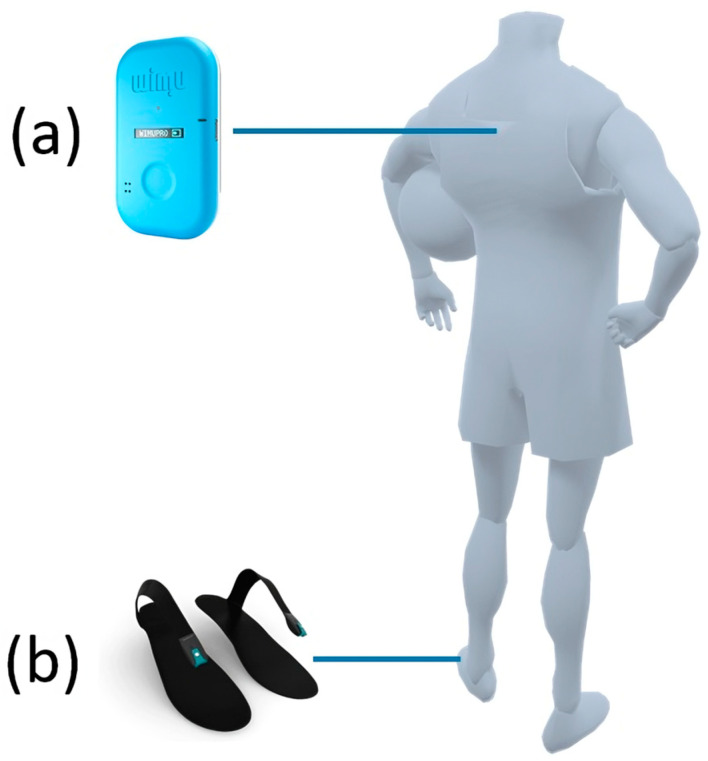
Sensor placement: WIMU PRO^TM^ device (**a**) positioned between scapulae using specific tight-fitting vest; Loadsol smart insoles (**b**) placed inside sports shoes.

This system integrates various sensors: (i) GPS/LPS operating at 18 Hz; (ii) four 3D accelerometers with different scale backgrounds (±16 g, ±32 g, and ±400 g, respectively) and adjustable sampling rates (ranging from 10 to 1000 Hz); (iii) three 3D gyroscopes (± 500 °/s, ± 2000 °/s, ± 4000 °/s full-scale output range) operating at a sampling rate of 1000 Hz; (iv) a 3D magnetometer; and (v) a barometer. The sensor was placed between the players’ scapulae using a specific tight vest (Figure 1a).

Prior to placement, the calibration and synchronization of the inertial devices were meticulously executed in accordance with the manufacturer’s guidelines.

The device was activated outdoors, ensuring minimal signal interference from nearby structures. As is consistent with the recommendations of Maddison and Ni Mhurchu [30], all devices were activated 30 minutes before data collection to allow the acquisition of satellite signals and synchronize the GPS clock with the satellite’s atomic clock [31]. To avoid inter-unit errors, each subject wore the same GPS device throughout the study [32].

Additionally, participants were equipped with smart insoles (Loadsol^®^) of appropriate size at the onset of the session (Figure 1b). These smart insoles contain a single capacitive force sensor along their length. The calibration of the smart insoles was conducted in accordance with the protocol outlined by Peebles et al. [33]. The resolution of the smart insoles was set to 1 N to allow for a force range of 0 to 3000 N with a sampling frequency of 200 Hz.

### 2.4. Data Analysis

The physical data obtained through the WIMU PRO™ device underwent initial analysis utilizing the SPRO™ software (version 977; RealtrackSystems S.L., Almeria, Spain). Triaxial accelerations, running stride parameters (CT, FT, K_vert_, and foot in contact with the ground (R/L)) and GPS speed were extracted from the WIMU software version 2.0.0.1 and exported in .csv format files.

The dataset obtained from the smart insoles was exported and reworked in Python version 3.10.10. The smart insoles’ data were filtered using a fifth-order Butterworth low-pass filter with a cut-off frequency of 40 Hz to minimize noise artifacts. A thresholding algorithm was implemented to analyze ground reaction force (GRF) data estimated using the smart insole. Specifically, foot strike and toe-off were identified using a threshold set at 10% of body weight [34], which represents the lowest threshold value that effectively mitigated the occurrence of false step identifications. From this process, we identified which foot was in contact with the ground and saved this information in the dataset. After extracting foot strike and toe-off timings, contact time (CT) and flight time (FT) were calculated for each stride. K_vert_ was calculated according to Morin’s method [9], which achieved high precision (R-squared = 0.97) in estimating K_vert_ from mass, flight time, and contact time. This sine-wave approach accurately estimates leg stiffness, assuming the force during contact time follows a sinusoidal function.

**Figure 2:**
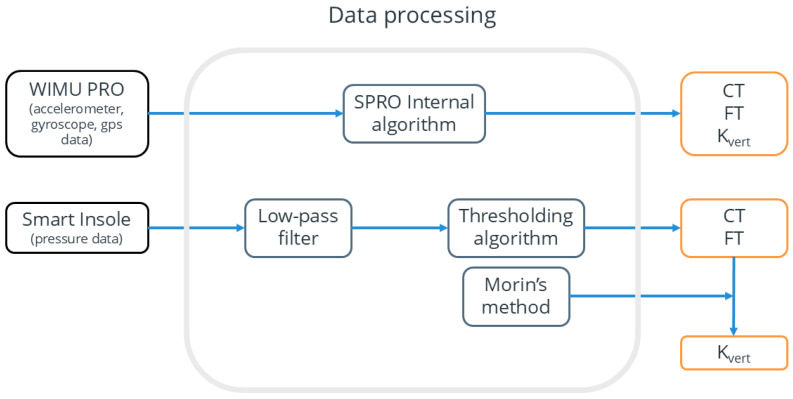
**The data processing pipeline for extracting contact time (CT), flight time (FT), and vertical stiffness (K_vert_) from both wearable systems.** The figure illustrates the data processing workflows for the WIMU PRO and smart insole devices. The WIMU PRO utilizes the SPRO internal algorithm to directly compute CT, FT, and K_vert_. For the smart insole, the raw data undergoes a low-pass filter before proceeding through a thresholding algorithm to determine CT and FT. Morin’s method is then applied to derive K_vert_ based on these time parameters. Both devices generate comparable output metrics (CT, FT, K_vert_) via distinct processing steps.

The data processing pipeline for both devices is illustrated in Figure 2, which outlines the steps involved in processing the raw data from both wearable systems.

At the end of this process, we obtained two datasets structured as follows:

- **WIMU PRO dataset**: Contains timestamps, accelerometer data, GPS data, contact time (CT), flight time (FT), vertical stiffness (K_vert_), and foot in contact with the ground.
- **Smart insole dataset**: Includes timestamps, ground reaction force (GRF) data from both feet, contact time (CT), flight time (FT), vertical stiffness (K_vert_), and foot in contact with the ground.

The original timestamps were preserved in both datasets to enable synchronization between the devices. The data from the two systems were synchronized using a common clock to ensure precise temporal alignment between the signals. During post-processing, the data were reviewed to assess the effectiveness of the synchronization and verify proper alignment of the signals. Specifically, the timestamps in the smart insole dataset were matched with those in the WIMU PRO dataset. The smart insole dataset provided the ground truth for identifying strides eligible for analysis, excluding those with walking patterns such as double support phases. For each eligible stride in the smart insole dataset, the corresponding stride in the WIMU PRO dataset was identified if it fell within a 30 ms temporal window and matched the side of the measurement (left or right). When these conditions were met, the data from the WIMU PRO were synchronized with the smart insole dataset, ensuring a unified dataset for subsequent analysis.

All the strides were classified based on GPS speed into 4 running activities: jog (7.1–14.3 km/h), run (14.4–19.7 km/h), high-speed run (19.8–24.9 km/h), and sprint (>25 km/h). To correct the K_vert_ values obtained from the WIMU device, a multivariate linear regression analysis was performed using an SPSS model. This analysis aimed to align the WIMU measurements more closely with those of the smart insoles.

Due to the fact that K_vert_ is highly dependent on running velocity [35,36,37], a variable that takes this variable into account has been added.

The regression formula applied was as follows:

(1) $$K_{vert\;Insole}\;=\;C_{1}\;*\;K_{vert\;WIMU\;}+\;C_{2}\;*\;Velocity\;+\;C_{3}$$

We determined the parameters using the mean squared error in leave-one-out cross-validation (LOOCV) [38]. We preferred to use LOOCV due to its property of providing an approximately unbiased estimation of the error [39]. In this method, one of the n participants was chosen for the test set in LOOCV, while the other *n* − 1 participants served as the training set. This process was repeated for all *n* participants. K_vert_ corrected is derived from the concatenation of the *n* test sets. The three coefficients are presented as the mean and standard deviation (SD) of the *n* algorithms, calculated using LOOCV.

### 2.5. Statistical Analysis

The statistical analysis was performed in IMB SPSS Statistics version 25.0 (SPSS Inc., Chicago, IL, USA) for the Windows statistical software package (the analysis was performed using SPSS software version 25.0 on a computer running Windows 11). The sample was initially described and characterized using descriptive statistics. The Shapiro–Wilk test was used to assess the assumption of normality. Afterward, the Wilcoxon test was used to compare the medians of the two devices at different running speeds. Non-parametric statistics were selected because they are ideal when data do not meet the assumptions of normality, independence, or homoscedasticity. They are also flexible, robust to non-normal data, and appropriate for small sample sizes. The temporal parameters were also evaluated using Pearson correlation coefficient, Cronbach’s alpha, and intra-class correlation (ICC(3,1)) statistics. While the Pearson correlation coefficient measures linear relationships between variables, Cronbach’s alpha evaluates the reliability of instruments, and the ICC(3,1) evaluates the agreement between methods, particularly when considering a single measure from a two-way mixed model. Each test ensures that different aspects of the data are examined for accurate and reliable results. All tests were performed for every running activity. The results were considered significant when *p* < 0.05.

## 3. Results

### 3.1. Temporal Parameters Description

The temporal parameter distributions were examined to compare the performance of WIMU with smart insole devices. To ensure accuracy, temporal parameter values equal to zero, caused by WIMU sensor detection failures, were excluded. Of the 1605 steps recorded, 1518 were retained for further analysis, resulting in only 5.5% of the steps being discarded as invalid. Table 1 presents the distribution of temporal parameters for both smart insoles and WIMU devices across different running speeds.

For CT, the WIMU consistently shows higher median values across all running speeds compared to smart insoles. In terms of FT, the WIMU generally shows lower median values compared to smart insoles. The interquartile ranges (IQRs) for FT reveal that WIMU’s measurements are less variable at higher speeds compared to the smart insoles.

K_vert_ measurements show that the WIMU records significantly lower median values across all running speeds compared to smart insoles. The IQRs indicate that the smart insoles have a wider range of K_vert_ values, suggesting greater variability in their measurements.

### 3.2. Statistical Results

Overall, the data indicates that the smart insoles tend to measure shorter contact times, longer flight times, and higher K_vert_ values compared to WIMU across all tested running speeds. And, indeed, the Wilcoxon *p*-values indicate significant differences (*p* < 0.001) between the smart insoles and WIMU measurements for all parameters and activities, as shown in Table 2. Additionally, a specific post hoc power analysis was performed for the outcomes of the present study. The mean power across all comparisons was calculated as 0.81.

#### 3.2.1. Contact Time (CT)

The ICC indicates moderate reliability for jogging (ICC = 0.542) and high-speed running (ICC = 0.478), but lower reliability for running (ICC = 0.313) and sprinting (ICC = 0.208), with the latter’s confidence interval including negative values. The Pearson correlation coefficients for CT are moderate for jogging (0.600) and high-speed running (0.512), weaker for running (0.317), and lowest for sprinting (0.280), although the correlation for sprinting is not statistically significant (*p* = 0.054). Cronbach’s Alpha values show higher internal consistency for jogging (0.703) and high-speed running (0.647) compared to running (0.477) and sprinting (0.345).

#### 3.2.2. Flight Time (FT)

The ICC values suggest low to moderate reliability, with the highest value for running (ICC = 0.383) and the lowest for sprinting (ICC = 0.205), again reflecting poor agreement for sprinting. Pearson correlation coefficients for FT are moderate for jogging (0.324) and running (0.386), but lower for high-speed running (0.274) and sprinting (0.309), though all are statistically significant except for sprinting. Cronbach’s Alpha values indicate higher consistency for running (0.554) and jogging (0.486) compared to high-speed running (0.426) and sprinting (0.341).

#### 3.2.3. Vertical Stiffness (K_vert_)

The ICC values indicate moderate reliability for jogging (0.386) and high-speed running (0.377), but lower reliability for running (0.267) and very low reliability for sprinting (0.161). The Pearson correlation coefficients are relatively strong for jogging (0.690) and high-speed running (0.600), moderate for running (0.458), and weak for sprinting (0.177), with the latter not being statistically significant (*p* = 0.228). Cronbach’s Alpha values suggest moderate internal consistency for jogging (0.557) and high-speed running (0.548), but are lower for running (0.421) and sprinting (0.278).

### 3.3. Linear Regression Analysis

The regression formula applied for the linear regression correction was as follows:

$$K_{vert\;Insole}\;=\;C_{1}\;*\;K_{vert\;WIMU\;}+\;C_{2}\;*\;Velocity\;+\;C_{3}$$

The parameters exerted from the LOOCV model are presented as follows (mean ± SD):

- *C*_1_=1.038 ±0.055: The coefficient for the WIMU-measured K_vert_.
- *C*_2_ = 2.435 ± 0.167 $\left[\frac{MN}{h}\right]$: The coefficient for the velocity factor.
- *C*_3_$=-24.545\;\pm 3.553\;[\frac{kN}{m}]$: The intercept of the regression equation.

The regression analysis indicates that the model explains a moderate proportion of the variance in the dependent variable (R-squared = 0.451). The model is highly significant (F-statistic *p*-value < 0.0001), and the RMSE values suggest reasonable predictive accuracy. The coefficients for the predictors are all significant, with low *p*-values and small standard errors. All the results of the regression analysis are reported in Table 3.

**Figure 3:**
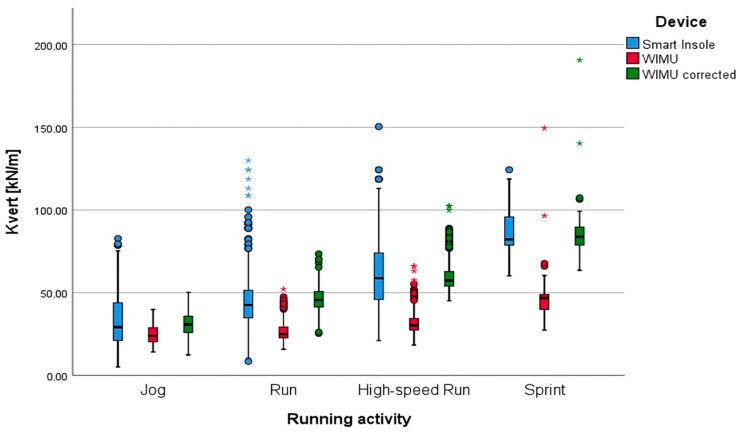
Boxplot of vertical stiffness (expressed in kilonewton per meter). Mild outliers—values beyond 1.5 × IQR below Q_1_ or above Q_3_—are shown as circles. Extreme outliers, exceeding 3.0 × IQR, are marked with asterisks.

The boxplot in Figure 3 shows the distribution of K_vert_ across all running activities. During jogging, the K_vert_ values measured by WIMU are lower compared to the smart insoles, with a percentage difference of approximately 29.3%. However, the WIMU corrected values are higher than the original WIMU readings and much closer to the gold standard, showing a percentage difference of only 3.7%. In fact, the Wilcoxon test result shows no differences between WIMU corrected and smart insoles with a *p*-value of 0.3999, as shown in Table 4.

During running, the chart indicates that WIMU values are significantly lower than the smart insoles, with a percentage difference of about 40.5%. After correction, the values increase and are much closer to the gold standard, with the percentage difference reduced to 3.7%, though the results remain statistically significant with a *p*-value of <0.001.

For high-speed running, the correction brings the values much closer to the gold standard, reducing the percentage difference from approximately 48.5% with WIMU to just 2.5% with WIMU corrected. However, the difference remains statistically significant with a *p*-value of 0.037.

In sprinting, WIMU values are low with greater variance, but the corrected values are much higher and closer to the gold standard, with reduced variance. The median stiffness for WIMU during sprinting is 45.6 kN/m (IQR = 12.7 kN/m), while the WIMU corrected shows a median of 84.1 kN/m (IQR = 12.8 kN/m), with the gold standard at 82.2 kN/m. The Wilcoxon test shows no differences between the WIMU corrected and smart insoles with a *p*-value of 0.857.

## 4. Discussion

The present study focused on evaluating K_vert_ during running and its intricate correlation with various biomechanical, neuromuscular, and metabolic manifestations of fatigue, as explored extensively in previous research [35,36,37,40,41,42,43]. By comparing the WIMU algorithm with the smart insoles, significant findings that contribute to this dynamic field were uncovered. This study has provided greater clarity as it evaluated a parameter estimated using a single accelerometer, comparing it to the gold standard. While the literature has validated the WIMU device primarily for estimating player positioning [26] and parameters related to the magnitude of acceleration (e.g., PlayerLoad) [27], it is important to note that this validation does not extend to the estimation of K_vert_. The WIMU algorithm for K_vert_ estimation, which relies on data collected from a single device, was compared with the precise measurements of smart insoles, considered the gold standard. This comparison allowed us to assess the accuracy and reliability of the WIMU algorithm, offering new insights into the use of less complex and potentially more accessible devices for monitoring biomechanical parameters.

The analysis of K_vert_ from ten subjects revealed that the WIMU system consistently underestimated the K_vert_ values when compared to the gold standard. This underestimation highlights the importance of accurate measurement tools in assessing biomechanical parameters, especially as running intensity increases. The data demonstrated clear and consistent patterns across different running activities, demonstrating that variations in CT and FT are intricately linked with changes in K_vert_. The findings from the smart insoles used in this study align with the results proposed by Morin et al. [9], reinforcing the validity of our results. Specifically, the K_vert_ data collected during this study corroborates the existing literature, providing further evidence that the observed relationships between CT, FT, and K_vert_ are robust and consistent with previously established scientific findings. This agreement with prior research underscores the reliability of our data and supports the broader applicability of our results. As runners transition from jogging to sprinting, the decrease in CT coupled with an increase in K_vert_ reflects the body’s adaptation to generate greater force over shorter ground contact periods. This phenomenon underscores the importance of lower limb stiffness in high-speed running performance and suggests that training programs aimed at improving K_vert_ could potentially enhance sprint performance. Furthermore, shorter FTs were found to correlate strongly with higher K_vert_ values across all types of activities [43]. These observations imply that changes in these temporal parameters can serve as reliable indicators of variations in the mechanical properties of the lower extremities, particularly K_vert_. Thus, the combination of temporal parameters and K_vert_ analyses can provide a more comprehensive understanding of biomechanical dynamics, which is crucial for optimizing athletic performance and preventing injuries.

Our regression model, incorporating velocity as a parameter, which has been shown to be deeply correlated with K_vert_ [35,36,40,41,44], demonstrated moderate success in correcting WIMU measurements. The improved accuracy post-correction represents a significant step forward in field-based biomechanical assessments. However, the varying correlation strengths across different running speeds suggest that there is still room for improvement, particularly in sprint measurements. The differences observed in K_vert_ values during high-speed running, even after correction, suggest that further improvements could be achieved by refining the algorithm. One approach could involve implementing machine learning models that dynamically adjust corrections based on running intensity, considering not only speed and acceleration but also other signals, such as trunk oscillation frequency.

The combination of temporal parameters and K_vert_ analysis provides a more comprehensive picture of running biomechanics than either measure alone. This approach could be particularly beneficial in understanding individual running styles, identifying potential injury risks, and tailoring training programs to optimize performance. Following the correction, the WIMU values were found to be much closer to those obtained using smart insoles across all running activities. Statistical tests indicated significant differences between the WIMU and smart insole measurements, underscoring the considerable improvement brought about by the correction. Factors such as the positioning of the WIMU device, potential movement artifacts during high-intensity activities, or limitations in the current algorithm might contribute to these inconsistencies.

The correction model developed in this study was designed to align IMU-based measurements with smart insole data but not to address the inherent limitations of the GPS systems integrated into WIMU devices. As a result, systemic issues with GPS technology may persist despite the model’s application. Widely used in sports, GPS technology is influenced by environmental factors such as stadium architecture, weather conditions, and the presence of spectators, which can negatively affect signal precision and reliability [45,46]. Future research to evaluate the robustness of the correction model under diverse practical constraints, such as environmental variability and device positioning, is necessary.

Study limitations include the small sample size of ten healthy subjects, variations in weather conditions during testing, and the self-selected running speeds. This study was designed as a preliminary feasibility investigation. While the sample size of ten participants, consisting solely of healthy male athletes, may limit generalizability, it allows for an initial evaluation of the proposed method under controlled conditions. Future research should evaluate the system’s performance with female athletes, different age groups, and varying skill levels. Although this study relied on smart insoles as the gold standard, additional validation of IMU-based K_vert_ estimation against laboratory-grade equipment such as force platforms could further enhance confidence in the results. However, it is important to note that variations in the testing setup, such as using treadmills or sensorized athletic tracks for validation, can influence K_vert_ estimation due to differences in running surfaces and conditions, potentially impacting the ecological validity of the results.

Despite these limitations, the statistical results still highlighted significant differences between the smart insole and WIMU measurements across all parameters and activities, with better performance observed in jogging compared to running and sprinting. This variability underscores the importance of considering these differences when conducting biomechanical analyses using these devices.

The research underscores the ongoing challenge of balancing accuracy with practicality in field settings. The need for easy-to-wear, fast, and accurate devices for testing entire teams remains a critical area for development in sports science.

Recent advancements in insole technology have facilitated the development of reliable systems. For example, the Loadsol insole was validated for assessing load-related and spatio-temporal metrics during functional activities, demonstrating good-to-excellent agreement with force platforms and instrumented treadmills [24]. Similarly, the XSENSOR X4 insole system has shown high reliability and validity in measuring vertical GRFs and center of pressure trajectories in hopping, walking, and running [25].

However, despite their accuracy in parameter estimation, these systems face scalability and practicality limitations in field settings due to logistical challenges, including the need for multiple devices and trained personnel for setup. In contrast, IMUs offer a cost-effective and practical solution, particularly in team sports, where scalability and ease of deployment are critical. The findings of this study demonstrate the potential of the IMU-based system as a simpler and more scalable alternative to smart insoles for monitoring vertical stiffness and related biomechanical parameters.

## 5. Conclusions

The improved accuracy of K_vert_ measurements has significant implications for sports science and athletic performance. It provides sports scientists with a more reliable metric to estimate player fatigue, potentially leading to more effective training regimens and injury prevention strategies. This advancement is particularly valuable in team sports settings, where quick and accurate biomechanical assessments of multiple athletes are essential. While our study demonstrates significant progress in wearable technology for biomechanical assessment, it also highlights the ongoing challenge of balancing accuracy with practicality in field settings. Future research should focus on further refining these measurement techniques and validating them across diverse populations and conditions.

In conclusion, this study underscores the importance of continually improving parameters obtained from wearable devices that are both easy to use and capable of rapid data collection for team-wide testing. The time- and labor-intensive nature of traditional biomechanical assessments often limits their application in team sports settings. By refining algorithms and enhancing the accuracy of devices like WIMU, we can move closer to a future where comprehensive biomechanical assessments are feasible for entire teams without compromising training time or requiring extensive technical expertise. This advancement would not only revolutionize performance monitoring but also contribute significantly to injury prevention strategies in team sports.

## Figures and Tables

**Table 1:** Temporal parameters distribution.

Temporal Parameter	Running Activities	Smart Insole		WIMU	
		Median	IQR	Median	IQR
Contact time [sec]	Jog	0.19	0.07	0.24	0.05
	Run	0.18	0.02	0.24	0.04
	High-Speed Run	0.16	0.03	0.22	0.03
	Sprint	0.13	0.02	0.18	0.04
Flight time [sec]	Jog	0.11	0.05	0.08	0.04
	Run	0.13	0.05	0.07	0.03
	High-Speed Run	0.12	0.03	0.06	0.01
	Sprint	0.11	0.02	0.05	0.03
Vertical stiffness [kN/m]	Jog	31.3	27.1	23.8	8.5
	Run	41.4	16.5	25	6.1
	High-Speed Run	55.1	29.6	30.3	6.5
	Sprint	82.2	28.7	45.6	12.7

**Table 2:** Statistical test results.

Temporal Parameter	Running activities	Wilcoxon *p*-Value	ICC	ICC CI95% min	ICC CI95% max	Pearson Coeff.	Pearson *p*-Value	Cronbach’s Alpha
Contact time [sec]	Jog	<0.001 *	0.542	0.39	0.67	0.600	<0.001 *	0.703
	Run	<0.001 *	0.313	0.25	0.37	0.317	<0.001 *	0.477
	High-Speed Run	<0.001 *	0.478	0.41	0.54	0.512	<0.001 *	0.647
	Sprint	<0.001 *	0.208	−0.08	0.46	0.280	0.054	0.345
Flight time [sec]	Jog	<0.001 *	0.321	0.13	0.49	0.324	0.001 *	0.486
	Run	<0.001 *	0.383	0.33	0.44	0.386	<0.001 *	0.554
	High-Speed Run	<0.001 *	0.270	0.19	0.35	0.274	<0.001 *	0.426
	Sprint	<0.001 *	0.205	−0.08	0.46	0.309	0.033 *	0.341
Vertical stiffness [kN/m]	Jog	<0.001 *	0.386	0.21	0.54	0.690	<0.001 *	0.557
	Run	<0.001 *	0.267	0.2	0.33	0.458	<0.001 *	0.421
	High-Speed Run	<0.001 *	0.377	0.3	0.45	0.600	<0.001 *	0.548
	Sprint	<0.001 *	0.161	−0.13	0.42	0.177	0.228	0.278

* The asterisk symbol (*) is utilized to highlight significant differences.

**Table 3:** Regression analysis statistics (SE = standard error).

Statistics	Mean	Standard Deviation	Min	Max
**R-squared**	0.451	0.037	0.359	0.496
**F-statistic**	582.084	78.082	391.822	687.140
**F-statistic *p*-value**	<0.001 *	<0.0001 *	<0.0001 *	<0.0001 *
**RMSE**	15.24	0.80	13.24	15.90
**C1 *p*-value**	<0.001 *	<0.0001 *	<0.0001 *	<0.0001 *
**C2 *p*-value**	<0.001 *	<0.0001 *	<0.0001 *	<0.0001 *
**C3 *p*-value**	<0.001 *	<0.0001 *	<0.0001 *	<0.0001 *
**SE C1**	0.061	0.006	0.055	0.076
**SE C2**	0.149	0.005	0.138	0.155
**SE C3**	2.42	0.07	2.30	2.57

* The asterisk symbol (*) is utilized to highlight significant differences.

**Table 4:** Statistical test results between smart insoles and WIMUs after linear regression correction.

Temporal Parameter	Running Activities	Wilcoxon *p*-Value
Vertical stiffness [kN/m]	Jog	0.399
	Run	<0.001 *
	High-Speed Run	0.037 *
	Sprint	0.857

* The asterisk symbol (*) is utilized to highlight significant differences.

## Data Availability

Data are available upon reasonable request.
